# Neighborhood Disadvantage, African Genetic Ancestry, Cancer Subtype, and Mortality Among Breast Cancer Survivors

**DOI:** 10.1001/jamanetworkopen.2023.31295

**Published:** 2023-08-30

**Authors:** Hari S. Iyer, Nur Zeinomar, Angela R. Omilian, Marley Perlstein, Melissa B. Davis, Coral O. Omene, Karen Pawlish, Kitaw Demissie, Chi-Chen Hong, Song Yao, Christine B. Ambrosone, Elisa V. Bandera, Bo Qin

**Affiliations:** 1 Cancer Epidemiology and Health Outcomes, Rutgers Cancer Institute of New Jersey, New Brunswick; 2 Department of Medicine, Rutgers Robert Wood Johnson Medical School, New Brunswick, New Jersey; 3 Department of Cancer Prevention and Control, Roswell Park Comprehensive Cancer Center, Buffalo, New York; 4 Institute of Genomic Medicine, Morehouse School of Medicine, Atlanta, Georgia; 5 Rutgers Cancer Institute of New Jersey, New Brunswick; 6 Cancer Epidemiology Services, New Jersey State Cancer Registry, New Jersey Department of Health, Trenton; 7 Department of Epidemiology and Biostatistics, SUNY Downstate Health Sciences University School of Public Health, Brooklyn, New York

## Abstract

**Importance:**

Racial disparities in breast cancer (BC) survival arise from multilevel causes, which may exert influence at different stages of BC progression. Clarifying the importance of genetic and social factors could help prioritize interventions.

**Objective:**

To jointly examine associations between African genetic ancestry, social environment, and mortality from any cause and BC in Black BC survivors.

**Design, Setting, and Participants:**

This population-based cohort study enrolled self-identified Black women aged 20 to 75 years with histologically confirmed BC from June 2005 to May 2019 and followed them up until death or censoring in September 2021. Participants lived in 10 New Jersey counties. Data were analyzed between December 2022 and April 2023.

**Exposures:**

A neighborhood socioeconomic status (nSES) index composed of census tract measures (education, income, wealth, employment status, and occupation) was linked to residential addresses at diagnosis. Percentage African ancestry was estimated using the ADMIXTURE program.

**Main Outcomes and Measures:**

Sequentially adjusted (age adjusted: age and interview year; fully adjusted: age adjusted with individual SES, lifestyle factors, and comorbidities) logistic regression models were fit to estimate associations with tumor subtypes (estrogen receptor–negative [ER^−^] vs estrogen receptor–positive [ER^+^]; triple-negative breast cancer [TNBC] vs luminal A), and Cox models were fit for associations with all-cause mortality (ACM) and breast cancer–specific mortality (BCSM). Models for BCSM were fit using Fine-Gray competing risks models, and robust standard errors were used to account for census tract–level clustering.

**Results:**

Among 1575 participants, median (IQR) African ancestry was 85% (76%-90%), and median (IQR) age was 55 (46-63) years. A 10–percentage point increase in African ancestry was associated with higher odds of ER^−^ vs ER^+^ (adjusted odds ratio [aOR], 1.08; 95% CI, 0.98-1.18) and TNBC vs luminal (aOR, 1.15; 95% CI, 1.02-1.31) tumors, but not with ACM or BCSM. A 1-IQR increase in nSES was associated with lower ACM (adjusted hazard ratio [aHR], 0.76; 95% CI, 0.63-0.93), and the HR for BCSM was less than 1 but not statistically significant (aHR, 0.81; 95% CI, 0.62-1.04) in age-adjusted models, but associations attenuated following further adjustment for potential mediators (individual SES, lifestyles, comorbidities).

**Conclusions and Relevance:**

In this cohort study of Black female BC survivors, higher African ancestry was associated with aggressive tumor subtypes. Compared with genetic ancestry, mediating pathways related to social environments may be more important for survival in these patients.

## Key Points

**Question:**

What are the relative strengths of associations of African genetic ancestry and neighborhood social environment with outcomes in Black breast cancer survivors?

**Findings:**

In this cohort study with 1575 Black female breast cancer survivors aged 20 to 75 years at diagnosis, African genetic ancestry was more strongly associated with tumor subtype than mortality. Associations between neighborhood socioeconomic status and mortality were attenuated following adjustment for potential mediators, including individual socioeconomic factors, lifestyle factors, and comorbidities.

**Meaning:**

In this study, African genetic ancestry was more strongly associated with tumor subtype, but social environment was more strongly associated with survival in Black breast cancer survivors.

## Teaser

This cohort study examines associations of African genetic ancestry and social environment with tumor subtype and all-cause and breast cancer–specific mortality among Black breast cancer survivors.

## Introduction

Breast cancer is a leading cause of cancer-related morbidity and mortality in US women, accounting for an estimated 297 800 new cases and 43 200 cancer deaths per year.^1^ Black or African American women (hereafter referred to as Black women) with breast cancer experience 41% higher mortality than White women.^2^ Black women are more likely to develop breast cancer at an early age^2^ and with more aggressive features, including triple-negative breast cancer (TNBC) characterized by aggressive tumors with mutations in key hormone receptors (estrogen receptor [ER]) and *ERBB2* (former *HER2*/neu).^3,4,5,6,7,8,9,10^ This excess risk of aggressive tumors in Black women may be partly explained by African ancestry–specific genetic variants, such as in the Duffy antigen receptor for chemokines (DARC/ACKR1),^11^ which may predispose carriers to lower immune response and higher inflammation as they age.^12,13,14,15,16,17^

Social and environmental factors also contribute to racial disparities in breast cancer.^18^ Structural racism,^19,20^ which leads to segregation and disinvestment in Black neighborhoods, may impact breast cancer tumor characteristics and survival through multiple pathways.^21,22,23,24^ Residential segregation influences access to care and neighborhood environments among Black women with breast cancer.^21,25^ Racial discrimination within the health care system creates barriers to accessing high-quality care for breast cancer in marginalized populations.^26,27^ Higher neighborhood socioeconomic status (nSES),^28^ characterized by a more favorable social environment and class, is associated with lower mortality in Black women with breast cancer^29,30,31^ and may be linked to lower risk of aggressive breast tumors.^32,33,34^ nSES may also influence the built environment,^32,35,36^ exposure to toxic chemicals,^37^ and social capital and community cohesiveness.^38^ nSES is also correlated with individual behaviors, such as smoking, obesity and physical inactivity, which are risk factors for breast cancer mortality.^36,39,40,41^

While available evidence suggests both genetic and environmental factors may explain excess mortality in Black women, few studies have incorporated them in their design and analysis.^42^ Failure to account for both genetic and nSES factors may lead to overestimates of their relative importance.^43,44^ To address these gaps, we examined joint associations of African ancestry (representing genetic influences) and nSES (representing environmental influences) on tumor aggressiveness and mortality in a large, population-based cohort of Black female breast cancer survivors.

## Methods

### Study Population and Design

Data were obtained from the Women’s Circle of Health Study^45^ and the Women’s Circle of Health Follow-Up Study (WCHFS), an ongoing cohort study of Black breast cancer survivors.^46^ Self-identified Black, English-speaking women diagnosed with breast cancer between June 2005 and May 2019, with histologically confirmed ductal carcinoma in situ or invasive breast cancer, aged 20 to 75 years at diagnosis, with no prior history of any cancer except nonmelanoma skin cancer, were eligible to participate in the study. Eligible women living in 10 counties of New Jersey were identified through the New Jersey State Cancer Registry (NJSCR). The institutional review boards at Rutgers University and Roswell Park Comprehensive Cancer Center approved the study protocol. Written consent was obtained from all participants. This study followed the Strengthening the Reporting of Observational Studies in Epidemiology (STROBE) reporting guideline.

Participants completed a baseline questionnaire within 10 months of diagnosis to report information on demographic characteristics, individual SES characteristics, lifestyle factors, comorbidities, and medical history. Anthropometric measures were taken by trained research staff during the home interview. Linkages with NJSCR data were used to collect information on diagnosis, hormone receptor status, clinical stage and grade, type of surgery, chemotherapy, radiation therapy, and endocrine therapy, complemented with information abstracted from medical records. Further details regarding the data collection procedures have been described previously.^40,46^ Of 1928 participants who completed the baseline survey, 1609 participants had genetic data available. We excluded 34 participants due to missing neighborhood and covariate data.

### African Ancestry

Participants provided a saliva sample at baseline using Oragene Self-Collection Kits (DNA Genotek, Inc). Specimens were shipped to the Roswell Park Data Bank and Biorepository for DNA extraction. NanoDrop and Qubit technologies were used for DNA quantification and quality assessment. DNA samples were stored at −80 °C prior to genotyping using the Illumina Multi-Ethnic Genotyping Array. For quality control, 2% blind duplicates and HapMap trio samples were included. Standard sample- and variant-level quality control procedures were performed. Genotype imputation was performed using the Trans-Omics for Precision Medicine (TOPMed) as a reference panel. Global percentage African, European, and Asian ancestry was estimated using the maximum likelihood-based ADMIXTURE method^47^ assuming 3 ancestral populations (k = 3) and data from the 1000 Genomes Project as references.

### nSES

The National Cancer Institute (NCI) nSES index was estimated and obtained from the NCI’s Surveillance Research Program.^48,49^ Participant residential addresses at diagnosis were geocoded by NJSCR and assigned to census tracts from the American Community Survey, with the 2013 nSES index (median year of diagnosis) used for linkage. The nSES index was calculated using a weighted education index,^50^ percentage unemployed, percentage working class, median household income, percentage below 150% of poverty line, median home value, and median rent. Higher scores indicated more neighborhood advantage.

### Tumor Subtypes

Information on breast cancer histopathological presentation, including immunohistochemical (IHC) status of ER, progesterone receptor (PR), and *ERBB2*, was obtained from medical records abstraction and pathology reports reviews, supplemented by linkages with the NJSCR. Breast tumors were classified into 3 IHC subtypes (luminal: ER-positive [ER^+^] and/or PR^+^ and *ERBB2*-negative (*ERBB2*^-^); *ERBB2*^+^; and TNBC: ER^−^, PR^-^, and *ERBB2*^-^). ER^−^ vs ER^+^ as well as TNBC vs luminal were examined to model associations of African ancestry with aggressive breast cancer.

### Mortality

Outcomes including date and cause of death were ascertained through linkage with NJSCR files. NJSCR updates vital status using various sources including state death certificates, hospital discharge files, Medicare and Medicaid files, Social Security Administration data, and the National Death Index. These data were complemented by active annual follow-up with participants. Deaths were updated through September 2021.

### Statistical Analysis

We summarized demographic, lifestyle, comorbidities, and clinical information across quartiles of African ancestry and nSES using frequency tables for categorical variables and medians with IQRs for continuous variables. Exposure was parameterized using a linear term (scaled to 10–percentage point increase for African ancestry, which was interpreted as a simultaneous 10–percentage point decrease in European ancestry, and scaled to IQR for nSES) and using quartiles with an ordinal test for trend. Divergence in survival curves by quartiles of African ancestry and nSES was assessed using log-rank tests.

We prepared a directed acyclic graph to assess confounding and mediation pathways for associations of African ancestry and nSES with breast cancer outcomes (eMethods and eFigure in Supplement 1).^51,52^ Models for ER^−^ breast cancer and tumor subtype were fit using logistic regression, while models for all-cause and breast cancer–specific mortality were fit using Cox proportional hazards models with study follow-up as the time scale, allowing the baseline hazard to vary between strata of age and interview year. Models for breast cancer–specific mortality were fit using Fine-Gray survival models with death due to non–breast cancer causes considered a competing risk.^53^

We fit regression models sequentially. Model 1, age, was adjusted for age (years, categorical: 21-40, 41-60, and 61-75 years) and interview year (categorical: 2005-2010, 2010-2015, and ≥2015); model 2, individual SES, was adjusted for education (categorical: high school or lower, some college, and college and postgraduate), mother’s education (binary: less than high school or high school or more), household income (categorical: <$25 000, $25 000-$69 999, ≥$70 000, and unknown), insurance (categorical: private, Medicare or Medicaid, uninsured, and other or missing), and marital status (categorical: married or living as married, widow, divorced or separated, and single or never married); model 3, comorbidities and lifestyle factors, was adjusted for history of hypertension, history of diabetes, smoking (categorical: never, former, and current), and body mass index (BMI [calculated as weight in kilograms divided by height in meters squared], categorical: <25, ≥25 to <30, and ≥30]); and model 4, clinical, was adjusted for breast cancer subtype (categorical: luminal, *ERBB2*^+^, TNBC, and unknown or missing), and chemotherapy receipt (binary: yes or no). We further considered parity, physical activity, alcohol use, and self-reported BMI prior to diagnosis, but inclusion did not change results and so they were not retained. Age and BMI were included as categorical; sensitivity analyses using continuous parameterization and 4-level categorization for BMI (<25, ≥25 to <30, ≥30 to <35, and ≥35) did not change results. In models for associations of nSES with breast cancer mortality, we interpreted effect estimates adjusted for individual SES and other factors as mediation, rather than confounding, reflecting multilevel conceptual models that treat upstream societal and neighborhood factors as occurring earlier in the causal process^51,54^ and because roughly 71% of participants in WCHFS lived at their residence for 5 or more years prior to diagnosis,^32^ while individual factors were collected at time of diagnosis (eMethods in Supplement 1). We did not report on nSES and tumor subtypes because results were published previously.^32^ We did not adjust for breast cancer subtype in models for ER^−^ breast cancer because subtype was assumed to lie on the causal path and selected parsimonious models for stage, subtype, and treatment due to high correlations between these measures. All models were fit using robust sandwich standard errors to account for Census tract-level clustering.

We further examined whether effect estimates for mortality varied by sociodemographic and clinical characteristics. Models were fit using multiplicative interaction terms between binary exposure variables (African ancestry: <85% or ≥85%; nSES: <9219 units or ≥9219 units; both dichotomized at median). In addition, as a sensitivity analysis, we repeated analyses excluding those who were not born in the United States, those with low-quality geocodes as assessed by NJSCR, and those with in situ breast cancer diagnoses. All tests were 2-sided with α = .05. Statistical analysis was performed using SAS version 9.4 (SAS Institute).

## Results

### Characteristics of the Study Population

Among 1575 participants included in the study, median (IQR) African ancestry was 85% (76%-90%) and median (IQR) age was 55 (46-63) years. WCHFS participants in the highest compared with lowest quartile of African ancestry were more likely to have never smoked, have higher BMI, have a high school graduate education or lower, have less than $25 000 in household income, reside in the lowest quartile of nSES index, and to present with ER^−^ tumors (Table 1). WCHFS participants in the highest (most advantaged) compared with lowest nSES quartile had higher individual SES and more favorable clinical characteristics at diagnosis (eTable 1 in Supplement 1).

**Table 1.** Characteristics of Black Women With Breast Cancer in the Women’s Circle of Health and Women’s Circle of Health Follow-Up Study, New Jersey, 2006 to 2020, Stratified by African Ancestry^a^

Characteristic	Participants, by quartile of African ancestry, No. (%)				
	All (N = 1575)	Quartile 1 (n = 396)	Quartile 2 (n = 390)	Quartile 3 (n = 398)	Quartile 4 (n = 391)
Ancestry, median (IQR), %					
African	85 (76-90)	68 (59-73)	81 (79-83)	87 (86-89)	94 (92-97)
Asian	1 (0-2)	1 (0-2)	1 (0-2)	1 (0-2)	0 (0-1)
European	14 (9-23)	31 (26-38)	18 (16-20)	12 (10-13)	5 (3-7)
Age at diagnosis, median (IQR), y	55 (46-63)	56 (46-63)	53 (45-61)	55 (47-63)	55 (47-64)
Interview year					
2006-2010	309 (20)	76 (19)	68 (17)	77 (19)	88 (23)
2011-2015	853 (54)	233 (59)	221 (57)	209 (53)	190 (49)
2016-2020	413 (26)	87 (22)	101 (26)	112 (28)	113 (29)
History of hypertension					
No	473 (30)	136 (34)	126 (32)	108 (27)	103 (26)
Yes	970 (62)	230 (58)	227 (58)	256 (64)	257 (66)
Missing	132 (8)	30 (8)	37 (9)	34 (9)	31 (8)
History of diabetes					
No	1078 (68)	280 (71)	272 (70)	273 (69)	253 (65)
Yes	365 (23)	86 (22)	81 (21)	91 (23)	107 (27)
Missing	132 (8)	30 (8)	37 (9)	34 (9)	31 (8)
Menopausal status	971 (62)	248 (63)	225 (58)	255 (64)	243 (62)
First-degree family history of breast cancer	281 (18)	77 (19)	60 (15)	75 (19)	69 (18)
Smoking status					
Never	932 (59)	206 (52)	222 (57)	242 (61)	262 (67)
Former	390 (25)	122 (31)	99 (25)	95 (24)	74 (19)
Current	253 (16)	68 (17)	69 (18)	61 (15)	55 (14)
Body mass index^b^					
<25.0	263 (17)	82 (21)	63 (16)	58 (15)	60 (15)
≥25.0 to <30.0	439 (28)	115 (29)	93 (24)	126 (32)	105 (27)
≥30.0	873 (55)	199 (50)	234 (60)	214 (54)	226 (58)
Alcohol consumption					
Nondrinker	940 (60)	233 (59)	223 (57)	239 (60)	245 (63)
≤3 drinks/wk	453 (29)	120 (30)	116 (30)	116 (29)	101 (26)
>3 drinks/wk	178 (11)	43 (11)	50 (13)	41 (10)	44 (11)
Missing	4 (<1)	0	1 (<1)	2 (1)	1 (<1)
Physical activity, MET-h/wk					
Quartile 1 (≤7.2)	384 (24)	85 (21)	98 (25)	99 (25)	102 (26)
Quartile 2 (7.3-21.2)	410 (26)	115 (29)	100 (26)	93 (23)	102 (26)
Quartile 3 (21.3-47.5)	382 (24)	90 (23)	106 (27)	98 (25)	88 (23)
Quartile 4 (≥47.6)	397 (25)	106 (27)	85 (22)	107 (27)	99 (25)
Not born in the US	263 (17)	61 (15)	24 (6)	41 (10)	137 (35)
Mother’s education					
Less than high school	475 (30)	88 (22)	95 (24)	137 (34)	155 (40)
High school or more	869 (55)	267 (67)	246 (63)	198 (50)	158 (40)
Unknown	231 (15)	41 (10)	49 (13)	63 (16)	78 (20)
Education					
High school graduate or lower	605 (38)	115 (29)	136 (35)	172 (43)	182 (47)
Some college	496 (31)	130 (33)	136 (35)	130 (33)	100 (26)
College and postgraduate	474 (30)	151 (38)	118 (30)	96 (24)	109 (28)
Household income					
<$25 000	477 (30)	98 (25)	115 (29)	134 (34)	130 (33)
$25 000-$69 999	541 (34)	120 (30)	121 (31)	158 (40)	142 (36)
≥$70 000	457 (29)	154 (39)	126 (32)	89 (22)	88 (23)
Unknown	100 (6)	24 (6)	28 (7)	17 (4)	31 (8)
Health insurance					
Private	862 (55)	233 (59)	215 (55)	220 (55)	194 (50)
Medicare or Medicaid	449 (29)	103 (26)	104 (27)	126 (32)	116 (30)
Uninsured	173 (11)	39 (10)	46 (12)	33 (8)	55 (14)
Other or missing	91 (6)	21 (5)	25 (6)	19 (5)	26 (7)
Marital status					
Married or living as married	584 (37)	168 (42)	137 (35)	129 (32)	150 (38)
Widowed	159 (10)	40 (10)	28 (7)	46 (12)	45 (12)
Divorced or separated	384 (24)	83 (21)	115 (29)	100 (25)	86 (22)
Single or never married	448 (28)	105 (27)	110 (28)	123 (31)	110 (28)
nSES (NCI index)					
Quartile 1	394 (25)	80 (20)	92 (24)	102 (26)	120 (31)
Quartile 2	396 (25)	90 (23)	109 (28)	110 (28)	87 (22)
Quartile 3	391 (25)	87 (22)	99 (25)	105 (26)	100 (26)
Quartile 4	394 (25)	139 (35)	90 (23)	81 (20)	84 (21)
Clinical characteristics					
Subtype					
Luminal	774 (49)	213 (54)	172 (44)	201 (51)	188 (48)
*ERBB2*^+^	280 (18)	63 (16)	81 (21)	69 (17)	67 (17)
TNBC	289 (18)	61 (15)	73 (19)	71 (18)	84 (21)
Unknown or missing	232 (15)	59 (15)	64 (16)	57 (14)	52 (13)
Grade					
1 (Well)	183 (12)	49 (12)	42 (11)	51 (13)	41 (10)
2 (Moderate)	610 (39)	149 (38)	150 (38)	150 (38)	161 (41)
3 (Poor)	695 (44)	173 (44)	177 (45)	177 (44)	168 (43)
Unknown	87 (6)	25 (6)	21 (5)	20 (5)	21 (5)
Stage					
0	280 (18)	76 (19)	72 (18)	74 (19)	58 (15)
I	574 (36)	150 (38)	154 (39)	133 (33)	137 (35)
II	504 (32)	111 (28)	113 (29)	139 (35)	141 (36)
III	170 (11)	51 (13)	37 (9)	41 (10)	41 (10)
IV	40 (3)	8 (2)	13 (3)	10 (3)	9 (2)
Unknown	7 (<1)	0	1 (<1)	1 (<1)	5 (1)
ER-negative	450 (29)	105 (27)	114 (29)	101 (25)	130 (33)
Treatment					
Surgical treatment					
No surgery	51 (3)	11 (3)	16 (4)	16 (4)	8 (2)
Lumpectomy	791 (50)	203 (51)	187 (48)	205 (52)	196 (50)
Mastectomy	733 (47)	182 (46)	187 (48)	177 (44)	187 (48)
Chemotherapy (yes)	864 (55)	210 (53)	207 (53)	220 (55)	227 (58)
Radiation therapy (yes)	1075 (68)	279 (70)	260 (67)	269 (68)	267 (68)
Endocrine therapy					
No	573 (36)	133 (34)	143 (37)	143 (36)	154 (39)
Yes	1001 (64)	263 (66)	247 (63)	255 (64)	236 (60)
Unknown or missing	1 (<1)	0	0	0	1 (<1)

Abbreviations: ER, estrogen receptor; MET, metabolic equivalent of task; NCI, National Cancer Institute; nSES, neighborhood socioeconomic status; TNBC, triple-negative breast cancer.

^a^ Wilcoxon or Kruskal-Wallis nonparametric tests were used to compare medians across quartiles of African ancestry; χ^2^ test of independence was used to compare prevalence.

^b^ Body mass index was calculated as weight in kilograms divided by height in meters squared.

### Associations Between African Ancestry and Tumor Characteristics

Each 10–percentage point increase in African ancestry was associated with 8% higher odds of ER^−^ vs ER^+^ tumor in age-adjusted models (adjusted odds ratio [aOR], 1.08; 95% CI, 0.98-1.18) (Table 2). Women in the highest compared with the lowest quartile experienced higher odds of ER^−^ tumors (aOR, 1.41; 95% CI, 1.05-1.90; *P *for trend = .08). Further adjustment for potential confounding by individual SES and lifestyles and comorbidities did not change these associations.

**Table 2.** Odds Ratios for Associations Between African Ancestry and Tumor Subtypes Among Black Women With Breast Cancer, New Jersey, 2006 to 2020

Model^a^	aOR (95% CI), by quartile of African Ancestry					*P* for trend
	Continuous^b^	Quartile 1	Quartile 2	Quartile 3	Quartile 4	
ER^−^** breast cancer (n = 1575)**						
ER^−^ cases, No. /ER^+^ cases, No.	NA	105/291	114/276	101/297	130/261	NA
Age^c^	1.08 (0.98-1.18)	1 [Reference]	1.13 (0.84-1.53)	0.95 (0.69-1.30)	1.41 (1.05-1.90)	.08
Age and individual SES^d^	1.07 (0.97-1.17)	1 [Reference]	1.11 (0.82-1.51)	0.92 (0.67-1.27)	1.38 (1.02-1.87)	.13
Age, individual SES, and lifestyles^e^	1.08 (0.98-1.18)	1 [Reference]	1.16 (0.85-1.57)	0.94 (0.68-1.31)	1.44 (1.06-1.95)	.09
Age, individual SES, lifestyles, and nSES^f^	1.08 (0.98-1.18)	1 [Reference]	1.15 (0.85-1.56)	0.94 (0.68-1.30)	1.43 (1.05-1.94)	.09
**TNBC vs luminal breast cancer (n = 1063)**						
TNBC cases, No./luminal cases, No.	NA	61/213	73/172	71/201	84/188	NA
Age^c^	1.14 (1.02-1.28)	1 [Reference]	1.49 (1.01-2.19)	1.25 (0.84-1.84)	1.63 (1.12-2.37)	.02
Age with individual SES^d^	1.15 (1.02-1.29)	1 [Reference]	1.49 (1.01-2.20)	1.22 (0.81-1.81)	1.66 (1.12-2.46)	.03
Age, individual SES, and lifestyles^e^	1.16 (1.02-1.31)	1 [Reference]	1.55 (1.05-2.29)	1.23 (0.82-1.85)	1.71 (1.15-2.56)	.02
Age, individual SES, lifestyles, and nSES^f^	1.15 (1.02-1.31)	1 [Reference]	1.55 (1.05-2.28)	1.22 (0.81-1.84)	1.70 (1.14-2.54)	.02

Abbreviations: aOR, adjusted odds ratio; ER^−^, estrogen receptor–negative; ER^+^, estrogen receptor–positive; NA, not applicable; nSES, neighborhood socioeconomic status; SES, socioeconomic status; TNBC, triple-negative breast cancer.

^a^ Models were fit using robust (sandwich) errors to account for census tract-level clustering.

^b^ Continuous measures are scaled to 10–percentage point increase for African ancestry, and interquartile range increase for nSES.

^c^ Models sequentially adjusted for age at diagnosis and interview year.

^d^ Models sequentially adjusted for education, mother’s education, household income, insurance, and marital status.

^e^ Models sequentially adjusted for history of hypertension, history of diabetes, smoking, and body mass index.

^f^ Models sequentially adjusted for nSES (IQR).

In age-adjusted models, a 10–percentage point increase in African ancestry was associated with 14% higher odds of TNBC vs luminal subtype (aOR, 1.14; 95% CI, 1.02-1.28) (Table 2). Comparing women in the highest with lowest quartile of African ancestry, there was 63% higher odds of TNBC (aOR, 1.63; 95% CI, 1.12-2.37; *P *for trend = .02) (Table 2). Further adjustment for individual SES (African ancestry quartile 4 vs 1: aOR, 1.66; 95% CI, 1.12-2.46; *P *for trend = .03) and lifestyles and comorbidities (African ancestry quartile 4 vs 1: aOR, 1.71, 95% CI, 1.15-2.56; *P *for trend = .02) did not change these associations. For both outcomes, after accounting for other factors, adjustment for nSES did not change results.

### Associations Between African Ancestry, nSES, and Mortality

There were 277 all-cause deaths over 11 460 person-years of follow-up. Survival curves were similar across quartiles of African ancestry (*P* = .45), but curves diverged across quartiles of nSES, with higher survival among women in the most compared with least advantaged quartile of nSES (*P* = .009) (Figure).

**Figure.**
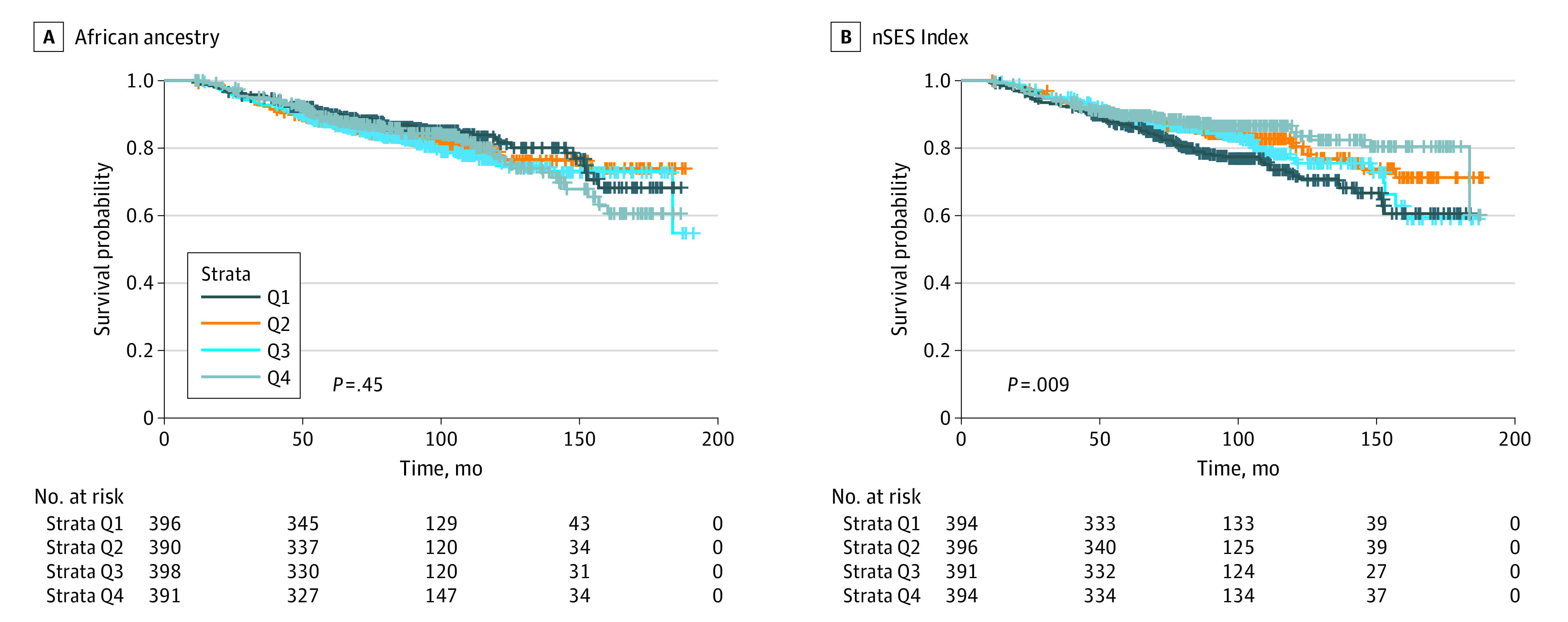
Kaplan-Meier Survival Curves for All-Cause Mortality by Quartiles (Q) of African Ancestry and Neighborhood Socioeconomic Status (nSES) in 1575 Black Women with Breast Cancer, New Jersey, 2006 to 2020 *P* value for log-rank test.

Higher percentage of African ancestry was associated with a 5% higher rate of all-cause mortality in age-adjusted models (10–percentage point increase: adjusted hazard ratio [aHR], 1.05; 95% CI, 0.96-1.16), although this was not statistically significant (Table 3). Additional adjustment for individual SES, nSES, and lifestyles and comorbidities did not change associations.

**Table 3.** Hazard Ratios for Associations Between African Ancestry, nSES, and Mortality Among Black Women With Breast Cancer, New Jersey, 2006 to 2020

Model^a^	aHR (95% CI)					*P* for trend
	Continuous^b^	Quartile 1	Quartile 2	Quartile 3	Quartile 4	
**All-cause mortality**						
African ancestry						
Deaths, No./person-years	NA	61/2967	68/2820	75/2813	73/2858	NA
Age^c^	1.05 (0.96-1.16)	1 [Reference]	1.24 (0.86-1.78)	1.34 (0.94-1.90)	1.24 (0.89-1.73)	.14
Age and individual SES^d^	1.01 (0.92-1.11)	1 [Reference]	1.18 (0.82-1.71)	1.20 (0.84-1.72)	1.09 (0.77-1.55)	.53
Age, individual SES, and lifestyles^e^	1.02 (0.93-1.12)	1 [Reference]	1.17 (0.80-1.71)	1.26 (0.88-1.81)	1.11 (0.78-1.59)	.43
Age, individual SES, lifestyles, and treatment^f^	1.02 (0.93-1.12)	1 [Reference]	1.18 (0.81-1.72)	1.31 (0.91-1.88)	1.10 (0.77-1.57)	.44
Age, individual SES, lifestyles, treatment, and nSES^g^	1.02 (0.93-1.12)	1 [Reference]	1.17 (0.80-1.71)	1.30 (0.91-1.87)	1.09 (0.76-1.57)	.45
nSES						
Deaths, No./person-years	NA	91/2897	66/2888	68/2774	52/2901	NA
Age^c^	0.76 (0.63-0.93)	1 [Reference]	0.76 (0.55-1.05)	0.80 (0.58-1.09)	0.59 (0.42-0.83)	.01
Age and individual SES^d^	0.89 (0.72-1.09)	1 [Reference]	0.81 (0.58-1.12)	0.92 (0.67-1.26)	0.76 (0.53-1.08)	.21
Age, individual SES, and lifestyles^e^	0.91 (0.74-1.12)	1 [Reference]	0.82 (0.59-1.15)	0.94 (0.68-1.31)	0.79 (0.55-1.14)	.32
Age, individual SES, lifestyles, and treatment^f^	0.96 (0.78-1.19)	1 [Reference]	0.88 (0.63-1.22)	1.00 (0.72-1.40)	0.88 (0.61-1.28)	.64
Age, individual SES, lifestyles, treatment, and African ancestry^g^	0.97 (0.78-1.19)	1 [Reference]	0.88 (0.63-1.22)	1.00 (0.72-1.40)	0.88 (0.61-1.28)	.66
**Breast cancer–specific mortality** ^h^						
African ancestry						
Deaths, No./person-years	NA	37/2969	35/2820	44/2813	35/2858	NA
Age^c^	0.99 (0.87-1.13)	1 [Reference]	0.96 (0.60-1.54)	1.19 (0.75-1.87)	0.96 (0.60-1.55)	.87
Age and individual SES^d^	0.96 (0.84-1.08)	1 [Reference]	0.91 (0.56-1.48)	1.08 (0.68-1.73)	0.84 (0.51-1.38)	.68
Age, individual SES, and lifestyles^e^	0.97 (0.85-1.10)	1 [Reference]	0.89 (0.54-1.45)	1.12 (0.70-1.79)	0.85 (0.51-1.41)	.76
Age, individual SES, lifestyles, and treatment^f^	0.95 (0.84-1.08)	1 [Reference]	0.86 (0.52-1.40)	1.09 (0.68-1.74)	0.82 (0.50-1.35)	.64
Age, individual SES, lifestyles, treatment, and nSES^g^	0.95 (0.84-1.08)	1 [Reference]	0.86 (0.52-1.41)	1.09 (0.68-1.74)	0.82 (0.50-1.35)	.65
nSES						
Deaths, No./person-years	NA	47/2897	37/2888	34/2774	33/2901	NA
Age^c^	0.81 (0.62-1.04)	1 [Reference]	0.78 (0.50-1.21)	0.77 (0.50-1.21)	0.71 (0.46-1.11)	.20
Age and individual SES^d^	0.94 (0.72-1.23)	1 [Reference]	0.84 (0.54-1.31)	0.91 (0.58-1.44)	0.93 (0.59-1.49)	.92
Age, individual SES, and lifestyles^e^	0.96 (0.73-1.27)	1 [Reference]	0.84 (0.53-1.32)	0.93 (0.59-1.48)	0.96 (0.60-1.56)	.97
Age, individual SES, lifestyles, and treatment^f^	1.03 (0.78-1.37)	1 [Reference]	0.89 (0.57-1.39)	0.99 (0.61-1.59)	1.10 (0.67-1.80)	.60
Age, individual SES, lifestyles, treatment, and African ancestry^g^	1.03 (0.77-1.37)	1 [Reference]	0.89 (0.57-1.38)	0.99 (0.62-1.59)	1.09 (0.67-1.78)	.62

Abbreviations: aHR, adjusted hazard ratio; nSES, neighborhood socioeconomic status; SES, socioeconomic status.

^a^ Models were fit using robust (sandwich) errors to account for census tract-level clustering.

^b^ Continuous measures are scaled to 10–percentage point increase for African ancestry and 1-IQR increase for nSES.

^c^ Models sequentially adjusted for age at diagnosis and interview year.

^d^ Models sequentially adjusted for education, mother’s education, household income, insurance, and marital status.

^e^ Models sequentially adjusted for history of hypertension, history of diabetes, smoking, and body mass index.

^f^ Models sequentially adjusted for molecular subtype and chemotherapy.

^g^ Models sequentially adjusted for nSES (IQR increase) or African ancestry (10–percentage point increase).

^h^ Models fit using Fine-Gray competing risks proportional hazards models.

Women with higher nSES had lower rates of all-cause mortality in age-adjusted models (1-IQR increase: aHR, 0.76, 95% CI, 0.63-0.93). Comparing women in the highest with lowest quartile of nSES, there was a 41% lower rate of all-cause mortality (aHR, 0.59; 95% CI, 0.42-0.83; *P *for trend = .01). We observed attenuation of this association toward the null with further adjustment for individual SES, lifestyles and comorbidities, and treatment. A 1-IQR increase in nSES in fully adjusted models was not statistically significantly associated with all-cause mortality (aHR, 0.96; 95% CI, 0.78-1.19).

There were 151 deaths from breast cancer. In age-adjusted competing risk models, there were no clear patterns of association between African ancestry and breast cancer–specific mortality (1-IQR increase: aHR, 0.99; 95% CI, 0.87-1.13) (Table 3). Further adjustment did not change associations. Participants with higher nSES had lower breast cancer–specific mortality risk, but these associations were not statistically significant. Adjustment for individual SES sharply attenuated associations to the null (1-IQR increase: aHR, 0.96; 95% CI, 0.73-1.27). Further adjustment for lifestyles and comorbidities, as well as treatment, did not change associations. For both mortality end points, mutual adjustment did not change findings.

### Effect Modification and Sensitivity Analysis

Although no statistically significant multiplicative interactions were observed, women with both high African ancestry (≥85%) and unfavorable tumor characteristics generally experienced the highest all-cause and breast cancer–specific mortality (eTable 2 in Supplement 1). Similarly, women with low nSES and less favorable tumor characteristics generally experienced the highest all-cause mortality. For breast cancer–specific mortality, the strongest associations between tumor characteristics and mortality were observed in women with high nSES, although differences were generally not statistically significant. Results from models for associations of African ancestry with tumor characteristics (eTable 3 in Supplement 1) and mortality end points (eTable 4 in Supplement 1) were similar to main results following restrictions to Black women with invasive breast cancer, high-quality geocodes, and who were born in the United States.

## Discussion

In this large study of Black female breast cancer survivors in New Jersey, African ancestry was associated with higher odds of TNBC and ER^−^ breast cancers, independent of demographic characteristics, individual SES, behavioral factors, and comorbidities. However, higher percentage of African ancestry was not associated with mortality. We previously showed lower nSES (more disadvantage) was associated with higher odds of TNBC, particularly in neighborhoods with low proportions of Black residents.^32^ In the present study, Black women living in more advantaged neighborhoods experienced lower mortality in age-adjusted models. Associations between nSES and mortality attenuated following adjustment for potential mediators, including individual SES, behavioral factors, and comorbidities.^55,56,57^ Together, these results suggest that ancestry-related genetic factors may contribute to racial differences in tumor aggressiveness at diagnosis.^12^ However, modifiable environmental pathways involving nSES, individual SES, behaviors, and clinical management may be more important drivers of survival disparities.^4,26,55^

Few studies have examined multilevel associations between African ancestry, neighborhood factors, tumor characteristics, and survival in Black female patients with breast cancer. Goel and colleagues^42^ examined associations between African ancestry, nSES, and tumor characteristics of 58 Black and 236 White women with breast cancer in Florida. They found that African genetic ancestry was correlated with TNBC in unadjusted models, but this association attenuated to the null following adjustment for nSES. In a secondary analysis of 2239 Black men and women in the Prostate, Lung, Colorectal, and Ovarian cancer screening trial, associations between African genetic ancestry, nSES, and overall mortality found that although African ancestry was initially associated with higher all-cause mortality, this association attenuated following adjustment for lifestyles and comorbidities.^43^ Similarly, no associations were observed between African ancestry and cancer-related mortality.

Associations between nSES and mortality were attenuated following adjustment for individual SES, behaviors, and comorbidities, suggesting that these may be potential mediating pathways for nSES-related disparities in this study. A recent review of nSES in relation to cancer incidence and mortality found no clear evidence for associations between nSES and breast cancer survival, although results varied by race and ethnicity.^58^ Studies conducted in Southern states have found that Black women experience higher mortality independent of nSES.^59,60^ Studies of breast cancer mortality conducted exclusively in Black women, such as the prospective Black Women’s Health Study (BWHS), have found that more favorable nSES is associated with lower mortality.^31^ More favorable nSES was associated with higher incidence of ER^+^ tumors in the BWHS, but this association attenuated after adjustment for reproductive and other breast cancer risk factors.^61^ An earlier study in WCHFS found that lower nSES was associated with higher risk of TNBC.^32^ Of the studies that examined potential pathways using mediation analyses, many have found that behavioral factors, health care access, and individual SES can explain associations between nSES and mortality.^55^ Racial discrimination or stress-related pathways may also serve as mediators of associations between nSES and mortality.^62,63,64,65^ For example, neighborhood- and individual-level social and environmental stressors are correlated with numerous biological mechanisms involved in carcinogenesis, including inflammation, allostatic load, and epigenetic modification.^66,67,68,69^

### Limitations

This study has limitations. Our analyses were conducted in women with breast cancer who agreed to participate; thus, associations could partly reflect selection bias.^70^ However, prior work comparing characteristics of WCHFS participants with all Black women with breast cancer captured in the NJSCR found no major differences.^46^ While our results may not be generalizable to Black women everywhere,^71^ this study contributes evidence from one of the largest population-based cohorts of Black breast cancer survivors. African ancestry may serve as a proxy for ancestry-related causal variants that confer genetic susceptibility, and future work should examine specific genetic variants associated with African ancestry and breast cancer.^14^ Unmeasured confounding by residential self-selection, health care access, and discrimination could be a threat to validity. Future studies should incorporate specific measures of structural racism and interpersonal racism^19,63^ and more detailed information on health care access.^51^ However, we controlled for major confounding variables, including individual SES, behavioral risk factors, clinical factors, and treatment. We lacked power to investigate breast cancer–specific mortality and interactions between ancestry, nSES, and clinical characteristics. Future studies in populations exhibiting greater admixture and wider geographic areas are needed to confirm these findings.

## Conclusions

This study provides evidence that African genetic ancestry may be associated with tumor aggressiveness at diagnosis, while nSES and modifiable social, behavioral, and access factors may have a greater association with survival in Black women with breast cancer. Screening interventions focused on genetic susceptibility and population-based interventions focused on access, environmental, and behavioral factors may help narrow racial disparities in breast cancer.

## Notes

Supplement 1. **eMethods.** Conceptual Framework to Develop Causal Directed Acyclic Graph **eFigure.** Causal Directed Acyclic Graph Depicting Pathways That Drive Excess Risk of Aggressive Tumors and Mortality Among Black Women With Breast Cancer **eTable 1.** Characteristics of Black Women With Breast Cancer in the Women’s Circle of Health and Women’s Circle of Health Follow-up Study, New Jersey, 2006-2020, Stratified by Neighborhood Socioeconomic Status Index **eTable 2.** Hazard Ratios for Associations Between Clinical Predictors of Mortality in Breast Cancer Survivors, Stratified by African Ancestry and Neighborhood Socioeconomic Status in the Women’s Circle of Health Follow-up Study, New Jersey, 2006-2020 **eTable 3.** Odds Ratios for Associations Between African Ancestry and Tumor Subtypes Among Black Women With Breast Cancer, Restricted to High Quality Geocodes, Invasive Breast Cancer, US-Born, New Jersey, 2006-2020 **eTable 4.** Hazard Ratios for Associations Between African Ancestry, Neighborhood Socioeconomic Status, and Among Black Women With Breast Cancer, Restricted to High Quality Geocodes, Invasive Breast Cancer, US-born (n=1,051), New Jersey, 2006-2020

Supplement 2.
Data Sharing Statement
