# Supplementary material for: Neighborhood Disadvantage, African Genetic Ancestry, Cancer Subtype, and Mortality Among Breast Cancer Survivors
Source: JAMA Netw Open. 2023 Aug 30;6(8):e2331295. doi: 10.1001/jamanetworkopen.2023.31295 (PMC10469269; doi:10.1001/jamanetworkopen.2023.31295)
Supplement: Supplement 1. — eMethods. Conceptual Framework to Develop Causal Directed Acyclic Graph eFigure. Causal Directed Acyclic Graph Depicting Pathways That Drive Excess Risk of Aggressive Tumors and Mortality Among Black Women With Breast Cancer eTable 1. Characteristics of Black Women With Breast Cancer in the Women’s Circle of Health and Women’s Circle of Health Follow-up Study, New Jersey, 2006-2020, Stratified by Neighborhood Socioeconomic Status Index eTable 2. Hazard Ratios for Associations Between Clinical Predictors of Mortality in Breast Cancer Survivors, Stratified by African Ancestry and Neighborhood Socioeconomic Status in the Women’s Circle of Health Follow-up Study, New Jersey, 2006-2020 eTable 3. Odds Ratios for Associations Between African Ancestry and Tumor Subtypes Among Black Women With Breast Cancer, Restricted to High Quality Geocodes, Invasive Breast Cancer, US-Born, New Jersey, 2006-2020 eTable 4. Hazard Ratios for Associations Between African Ancestry, Neighborhood Socioeconomic Status, and Among Black Women With Breast Cancer, Restricted to High Quality Geocodes, Invasive Breast Cancer, US-born (n=1,051), New Jersey, 2006-2020 [file jamanetwopen-e2331295-s001.pdf]

# Supplemental Online Content

Iyer HS, Zeinomar N, Omilian AR, et al. Neighborhood disadvantage, African genetic ancestry, cancer subtype, and mortality among breast cancer survivors. *JAMA Netw Open.* 2023;6(8):e2331295. doi:10.1001/jamanetworkopen.2023.31295

**eMethods.** Conceptual Framework to Develop Causal Directed Acyclic Graph

**eFigure.** Causal Directed Acyclic Graph Depicting Pathways That Drive Excess Risk of Aggressive Tumors and Mortality Among Black Women With Breast Cancer

**eTable 1.** Characteristics of Black Women With Breast Cancer in the Women's Circle of Health and Women's Circle of Health Follow-up Study, New Jersey, 2006-2020, Stratified by Neighborhood Socioeconomic Status Index

**eTable 2.** Hazard Ratios for Associations Between Clinical Predictors of Mortality in Breast Cancer Survivors, Stratified by African Ancestry and Neighborhood Socioeconomic Status in the Women's Circle of Health Follow-up Study, New Jersey, 2006-2020

**eTable 3.** Odds Ratios for Associations Between African Ancestry and Tumor Subtypes Among Black Women With Breast Cancer, Restricted to High Quality Geocodes, Invasive Breast Cancer, US-Born, New Jersey, 2006-2020

**eTable 4.** Hazard Ratios for Associations Between African Ancestry, Neighborhood Socioeconomic Status, and Among Black Women With Breast Cancer, Restricted to High Quality Geocodes, Invasive Breast Cancer, US-born (n=1,051), New Jersey, 2006-2020

This supplemental material has been provided by the authors to give readers additional information about their work.

© 2023 Iyer HS et al. *JAMA Network Open.*

**eMethods. Conceptual Framework to Develop Causal Directed Acyclic Graph**

We developed a Directed Acyclic Graph (DAG) to guide selection of contextual and individual-level covariates in analyses estimating impacts of African genetic ancestry and nSES on tumor characteristics and mortality endpoints in WCHFS based on earlier work[1] and recommendations for developing DAGs to examine health disparities[2] (Supplementary Figure 1). DAGs serve two primary purposes: (1) visualize investigator assumptions regarding causal pathways that link measured variables in a data analysis, and (2) identify sources of bias[3].

If an arrow is drawn on the DAG between two variables, the investigator assumes causal pathways between those variables flowing in the direction of the arrow and statistically, the variables are correlated in the data. Absence of arrows between two variables means that the investigator assumes no causal path between them (and statistically, these variables are independent). Covariate adjustment is depicted on a DAG by drawing a box around a variable. When a variable is a common cause of two variables, or a descendant of two other variables and it has been adjusted for, it can create a spurious statistical association between those variables, or bias[4].

We drew a DAG including all potential factors (measured and unmeasured) that may be involved in causal relationships between the variables of interest (here, African genetic ancestry, nSES, tumor characteristics, and mortality). We included unmeasured historical influences of society, including social policies and environmental threats, all of which lead to geographic migration patterns that can influence health[5–10]. These factors can lead to racism (at societal or individual level)[11], and the subsequent development of socially assigned racial groups[10,12]. While self-identified race and ethnicity (SIRE) was the primary racial and ethnic construct used to classify women in this study, SIRE is increasingly understood to reflect the impacts of being socially assigned to one's racial/ethnic group[13], and so we added an arrow from socially assigned race and ethnicity to self-identified race and ethnicity.

In studies of Black women with breast cancer, SIRE is correlated with measured demographic variables (age, gender, sex), lifestyles (physical activity, alcohol use, body mass index, smoking), and comorbidities (hypertension, diabetes)[14–16]. An adult's health and socioeconomic status is a function of contextual (socioeconomic opportunities, political context, racial discrimination[17,18]) and individual (skills, educational attainment, flourishing[19]) factors. Because our study only collects measures in adulthood, we lack data on early life factors related to behaviors and lifestyles. We chose to include measures of Mother's educational attainment and country of birth to partially control for early life factors that may influence health outcomes of adult WCHFS participants.

Genetic ancestry is included in the DAG as a potential cause of adverse tumor characteristics and mortality[20,21]. Because we restrict our study population to Black/African American women, we induce selection bias if we fail to consider other socioeconomic, demographic, and lifestyle factors[1]. The pathways through which selection bias may arise for estimating effects of genetic ancestry on tumor characteristics and mortality are depicted in red.

*Interpretation of association of nSES with mortality adjusted for individual SES and other factors*

We did not collect data on nSES in early life, which would reflect consequences of structural racism and segregation on individual SES and related health impacts[10,18]. Assuming that individual SES (captured by educational attainment, income) reflects achievements prior to diagnosis, and nSES reflects neighborhood social environment at diagnosis, one could interpret models adjusting for individual SES as reflecting adjustment for confounding. However, we chose to interpret results from models for nSES and mortality adjusted for individual SES and other factors as mediation rather than confounding for two main

© 2023 Iyer HS et al. *JAMA Network Open.*

reasons. First, multilevel conceptual models that integrate neighborhood and individual factors that drive disparities view upstream societal and neighborhood factors as occurring earlier in the causal process[2,22]. This ordering reflects current understanding of how historical and ongoing social processes, such as structural racism, which drives residential segregation and differences in nSES across racial and ethnic groups[10,18]. Analysis of trends in individual and neighborhood-level SES across different racial and ethnic groups in the US reveals that Black individuals with higher individual income and educational attainment are less likely to reside in high SES neighborhoods compared to other racial and ethnic groups[23]. Second, in our study, individual-level SES and other factors were assessed at time of diagnosis. However, we previously found that 71% of the participants in the WCHFS had lived at their residence for at least five years[24]. This suggests possible influences of residential nSES on income, marital status, and insurance, and other factors.

Based on the literature on multilevel conceptual frameworks for studying health disparities and the design of our study, we chose as our primary interpretation that adjusting for individual SES and behaviors could reflect mediation, rather than control for confounding. Others have proposed DAGs for health disparities research that make similar assumptions to capture historical and ongoing social processes that impact health in marginalized communities[2].

## Supplementary Methods References


1. Iyer HS, Gomez SL, Cheng I, Rebbeck TR. Relative impact of genetic ancestry and neighborhood socioeconomic status on all-cause mortality in self-identified African Americans. PLOS ONE. 2022 Aug 29;17(8):e0273735.

2. Howe CJ, Bailey ZD, Raifman JR, Jackson JW. Recommendations for Using Causal Diagrams to Study Racial Health Disparities. Am J Epidemiol. 2022 Nov 19;191(12):1981–9.

3. Greenland S, Pearl J, Robins JM. Causal Diagrams for Epidemiologic Research. Epidemiology. 1999;10(1):37–48.

4. Hernán MA, Hernández-Díaz S, Robins JM. A structural approach to selection bias. Epidemiol Camb Mass. 2004 Sep;15(5):615–25.

5. Bryc K, Durand EY, Macpherson JM, Reich D, Mountain JL. The Genetic Ancestry of African Americans, Latinos, and European Americans across the United States. Am J Hum Genet. 2015 Jan 8;96(1):37–53.

6. Dai CL, Vazifeh MM, Yeang CH, Tachet R, Wells RS, Vilar MG, et al. Population Histories of the United States Revealed through Fine-Scale Migration and Haplotype Analysis. Am J Hum Genet. 2020 Mar 5;106(3):371–88.

7. Baharian S, Barakatt M, Gignoux CR, Shringarpure S, Errington J, Blot WJ, et al. The Great Migration and African-American Genomic Diversity. PLOS Genet. 2016 May 27;12(5):e1006059.

8. Martini R, Newman L, Davis M. Breast cancer disparities in outcomes; unmasking biological determinants associated with racial and genetic diversity. Clin Exp Metastasis. 2022 Feb 1;39(1):7–14.


© 2023 Iyer HS et al. *JAMA Network Open.*

9. Krieger N, Jahn JL, Waterman PD. Jim Crow and estrogen-receptor-negative breast cancer: US-born black and white non-Hispanic women, 1992–2012. Cancer Causes Control. 2017 Jan 1;28(1):49–59.

10. Bailey ZD, Krieger N, Agénor M, Graves J, Linos N, Bassett MT. Structural racism and health inequities in the USA: evidence and interventions. The Lancet. 2017 Apr 8;389(10077):1453–63.

11. Jones CP. Levels of racism: a theoretic framework and a gardener's tale. Am J Public Health. 2000 Aug;90(8):1212–5.

12. Williams DR, Lawrence JA, Davis BA. Racism and Health: Evidence and Needed Research. Annu Rev Public Health. 2019 01;40:105–25.

13. White K, Lawrence JA, Tchangalova N, Huang SJ, Cummings JL. Socially-assigned race and health: a scoping review with global implications for population health equity. Int J Equity Health. 2020 Feb 10;19(1):25.

14. Bandera EV, Qin B, Lin Y, Zeinomar N, Xu B, Chanumolu D, et al. Association of Body Mass Index, Central Obesity, and Body Composition With Mortality Among Black Breast Cancer Survivors. JAMA Oncol. 2021 Aug 1;7(8):1186–95.

15. Qin B, Kim K, Goldman N, Rundle AG, Chanumolu D, Zeinomar N, et al. Multilevel Factors for Adiposity Change in a Population-Based Prospective Study of Black Breast Cancer Survivors. J Clin Oncol. 2022 Jul 10;40(20):2213–23.

16. Zeinomar N, Qin B, Amin S, Lin Y, Xu B, Chanumolu D, et al. Association of Cigarette Smoking and Alcohol Consumption With Subsequent Mortality Among Black Breast Cancer Survivors in New Jersey. JAMA Netw Open. 2023 Jan 3;6(1):e2252371.

17. Chetty R, Hendren N, Katz L. The Effects of Exposure to Better Neighborhoods on Children: New Evidence from the Moving to Opportunity Project. Am Econ Rev. 2016;106(4).

18. Williams DR, Collins C. Racial Residential Segregation: A Fundamental Cause of Racial Disparities in Health - David R. Williams, Chiquita Collins, 2001. Public Health Rep [Internet]. 2001 Sep 1 [cited 2021 Jan 18]; Available from: http://journals.sagepub.com/doi/abs/10.1093/phr/116.5.404

19. VanderWeele TJ. On the promotion of human flourishing. Proc Natl Acad Sci. 2017 Aug;114(31):8148–56.

20. Rebbeck TR, Mahal B, Maxwell KN, Garraway IP, Yamoah K. The distinct impacts of race and genetic ancestry on health. Nat Med. 2022 May;28(5):890–3.

21. Dietze EC, Sistrunk C, Miranda-Carboni G, O'Regan R, Seewaldt VL. Triple-negative breast cancer in African-American women: disparities versus biology. Nat Rev Cancer. 2015 Apr;15(4):248–54.

22. Warnecke RB, Oh A, Breen N, Gehlert S, Paskett E, Tucker KL, et al. Approaching Health Disparities From a Population Perspective: The National Institutes of Health Centers for Population Health and Health Disparities. Am J Public Health. 2008 Sep;98(9):1608–15.

© 2023 Iyer HS et al. *JAMA Network Open.*

23. Massey DS, Tannen J. Segregation, Race, and the Social Worlds of Rich and Poor. In: Kirsch I, Braun H, editors. The Dynamics of Opportunity in America: Evidence and Perspectives [Internet]. Cham: Springer International Publishing; 2016 [cited 2023 Jun 22]. p. 13–33. Available from: https://doi.org/10.1007/978-3-319-25991-8_2

24. Qin B, Babel RA, Plascak JJ, Lin Y, Stroup AM, Goldman N, et al. Neighborhood social environmental factors and breast cancer subtypes among Black women. Cancer Epidemiol Biomark Prev. 2021 Feb;30(2):344–50.

© 2023 Iyer HS et al. *JAMA Network Open.*

**eFigure. Causal Directed Acyclic Graph Depicting Pathways That Drive Excess Risk of Aggressive Tumors and Mortality Among Black Women With Breast Cancer**
.

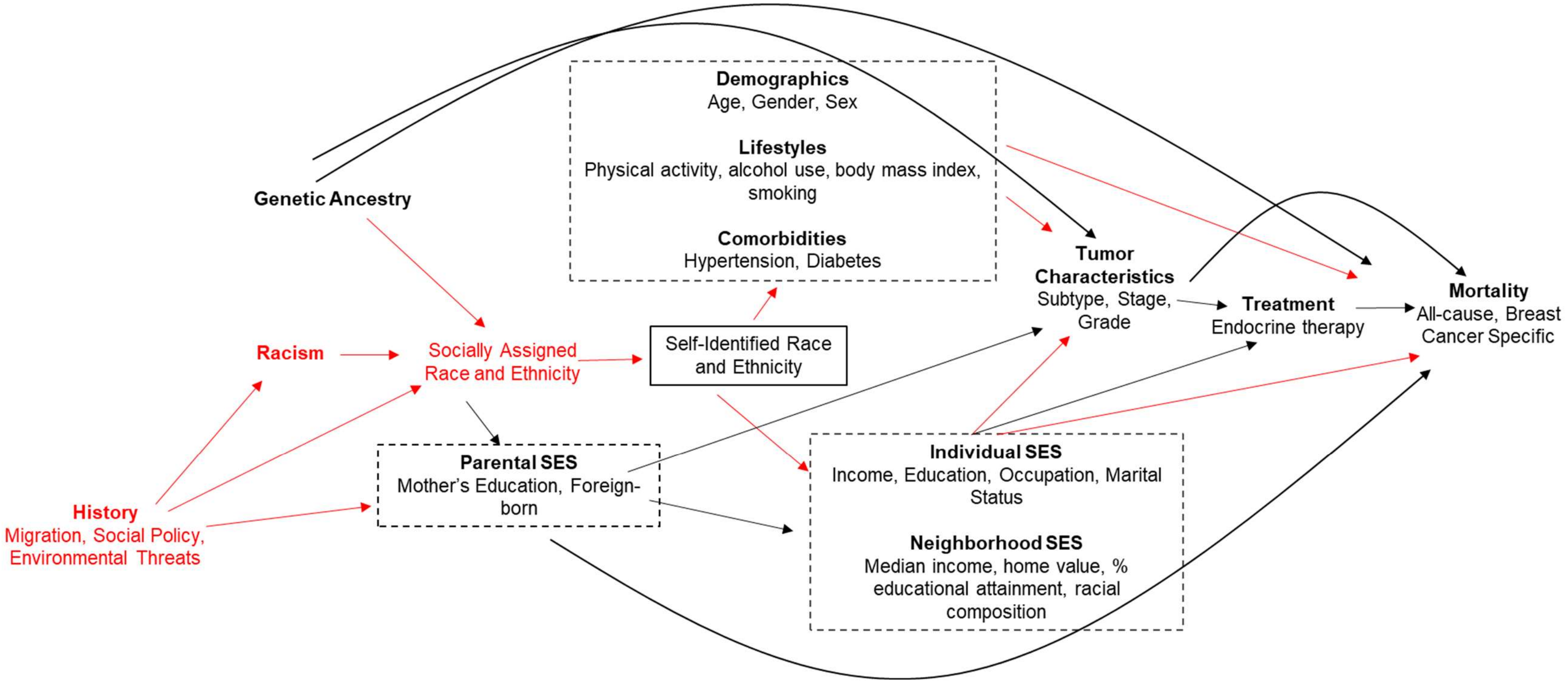


Legend: Arrows indicate hypothesized causal relationships between measured (black) and unmeasured (red) factors. Boxes with solid lines indicate restriction or adjustment; boxes with dotted lines indicate measures that are controlled for in the analysis. Red lines indicate non-causal (spurious) associations that can be resolved through adjustment.

© 2023 Iyer HS et al. *JAMA Network Open.*

**eTable 1. Characteristics of Black Women With Breast Cancer in the Women's Circle of Health and Women's Circle of Health Follow-up Study, New Jersey, 2006-2020, Stratified by Neighborhood Socioeconomic Status Index**

| | nSES (NCI Index) | | | | | |
|---|---|---|---|---|---|---|
| | **Total** | **Quartile 1** | **Quartile 2** | **Quartile 3** | **Quartile 4** | ***P***[a] |
| **N (%)** | 1,575 | 394 | 396 | 391 | 394 | |
| **Ancestry (percent), median [IQR]** | | | | | | |
| African | 85 [76, 90] | 86 [78, 91] | 85 [77, 89] | 85 [77, 91] | 82 [72, 89] | 0.050 |
| Asian | 1 [0, 2] | 1 [0, 1] | 1 [0, 2] | 0 [0, 1] | 0 [0, 2] | <.0001 |
| European | 14 [9, 23] | 13 [8, 21] | 14 [9, 21] | 14 [9, 21] | 16 [10, 27] | 0.32 |
| **Age at diagnosis (years), median [IQR]** | 55 [46, 63] | 55 [47, 63] | 54 [45, 62] | 56 [48, 64] | 54 [46, 61] | <.0001 |
| **Interview Year** | | | | | | 0.0049 |
| 2006-2010 | 309 (20) | 95 (24) | 84 (21) | 54 (14) | 76 (19) | |
| 2011-2015 | 853 (54) | 216 (55) | 210 (53) | 221 (57) | 206 (52) | |
| 2016-2020 | 413 (26) | 83 (21) | 102 (26) | 116 (30) | 112 (28) | |
| **History of hypertension** | | | | | | 0.014 |
| No | 473 (30) | 99 (25) | 117 (30) | 114 (29) | 143 (36) | |
| Yes | 970 (62) | 257 (65) | 248 (63) | 251 (64) | 214 (54) | |
| Missing | 132 (8) | 38 (10) | 31 (8) | 26 (7) | 37 (9) | |
| **History of diabetes** | | | | | | 0.19 |
| No | 1078 (68) | 259 (66) | 261 (66) | 278 (71) | 280 (71) | |
| Yes | 365 (23) | 97 (25) | 104 (26) | 87 (22) | 77 (20) | |
| Missing | 132 (8) | 38 (10) | 31 (8) | 26 (7) | 37 (9) | |
| **Menopausal status** | 971 (62) | 239 (61) | 241 (61) | 260 (67) | 231 (59) | 0.13 |
| **Family History of Breast Cancer** | 281 (18) | 80 (20) | 72 (18) | 66 (17) | 63 (16) | 0.42 |
| **Smoking Status** | | | | | | <.0001 |
| Never | 932 (59) | 214 (54) | 220 (56) | 236 (60) | 262 (67) | |
| Former | 390 (25) | 89 (23) | 101 (26) | 100 (26) | 100 (25) | |
| Current | 253 (16) | 91 (23) | 75 (19) | 55 (14) | 32 (8) | |
| **Body Mass Index, kg/m$^2$** | | | | | | 0.098 |
| <25.0 | 263 (17) | 66 (17) | 56 (14) | 63 (16) | 78 (20) | |
| ≥25.0 - <30.0 | 439 (28) | 96 (24) | 116 (29) | 106 (27) | 121 (31) | |
| ≥30.0 | 873 (55) | 232 (59) | 224 (57) | 222 (57) | 195 (49) | |
| **Alcohol consumption history at baseline** | | | | | | 0.021 |
| Non-drinker | 940 (60) | 249 (63) | 257 (65) | 220 (56) | 214 (54) | |
| ≤3 drinks per week | 453 (29) | 93 (24) | 97 (24) | 131 (34) | 132 (34) | |
| >3 drinks per week | 178 (11) | 51 (13) | 41 (10) | 39 (10) | 47 (12) | |
| Missing | 4 (0) | 1 (0) | 1 (0) | 1 (0) | 1 (0) | |
| **Physical activity, MET-hours/week** | | | | | | 0.060 |

© 2023 Iyer HS et al. *JAMA Network Open.*

| | nSES (NCI Index) | | | | | |
|---|---|---|---|---|---|---|
| | Total | Quartile 1 | Quartile 2 | Quartile 3 | Quartile 4 | *P*[a] |
| **N (%)** | 1,575 | 394 | 396 | 391 | 394 | |
| Quartile 1 (≤ 7.2) | 384 (24) | 122 (31) | 96 (24) | 83 (21) | 83 (21) | |
| Quartile 2 (7.3-21.2) | 410 (26) | 100 (25) | 108 (27) | 96 (25) | 106 (27) | |
| Quartile 3 (21.2-47.5) | 382 (24) | 85 (22) | 98 (25) | 100 (26) | 99 (25) | |
| Quartile 4 (≥ 48.0) | 397 (25) | 87 (22) | 94 (24) | 110 (28) | 106 (27) | |
| **Foreign-born** | 263 (17) | 55 (14) | 68 (17) | 62 (16) | 78 (20) | 0.39 |
| **Mother's Education** | | | | | | 0.0001 |
| Less than high school | 475 (30) | 138 (35) | 122 (31) | 117 (30) | 98 (25) | |
| High school or more | 869 (55) | 185 (47) | 211 (53) | 217 (56) | 256 (65) | |
| Unknown | 231 (15) | 71 (18) | 63 (16) | 57 (15) | 40 (10) | |
| **Education** | | | | | | <.0001 |
| High school graduate or lower | 605 (38) | 198 (50) | 183 (46) | 143 (37) | 81 (21) | |
| Some college | 496 (31) | 130 (33) | 122 (31) | 118 (30) | 126 (32) | |
| College and postgraduate | 474 (30) | 66 (17) | 91 (23) | 130 (33) | 187 (47) | |
| **Household income** | | | | | | <.0001 |
| <$25,000 | 477 (30) | 172 (44) | 139 (35) | 106 (27) | 60 (15) | |
| $25,000-$69,999 | 541 (34) | 145 (37) | 145 (37) | 143 (37) | 108 (27) | |
| ≥$70,000 | 457 (29) | 54 (14) | 90 (23) | 119 (30) | 194 (49) | |
| Unknown | 100 (6) | 23 (6) | 22 (6) | 23 (6) | 32 (8) | |
| **Health insurance** | | | | | | <.0001 |
| Private | 862 (55) | 170 (43) | 211 (53) | 224 (57) | 257 (65) | |
| Medicare/Medicaid | 449 (29) | 145 (37) | 118 (30) | 110 (28) | 76 (19) | |
| Uninsured | 173 (11) | 58 (15) | 46 (12) | 31 (8) | 38 (10) | |
| Other/missing | 91 (6) | 21 (5) | 21 (5) | 26 (7) | 23 (6) | |
| **Marital status** | | | | | | <.0001 |
| Married/living as married | 584 (37) | 111 (28) | 141 (36) | 150 (38) | 182 (46) | |
| Widowed | 159 (10) | 39 (10) | 44 (11) | 44 (11) | 32 (8) | |
| Divorced/separated | 384 (24) | 96 (24) | 100 (25) | 92 (24) | 96 (24) | |
| Single/never married | 448 (28) | 148 (38) | 111 (28) | 105 (27) | 84 (21) | |
| **Clinical Characteristics** | | | | | | |
| **Subtype** | | | | | | 0.089 |
| Luminal | 774 (49) | 196 (50) | 189 (48) | 195 (50) | 194 (49) | |
| Her2+ | 280 (18) | 70 (18) | 71 (18) | 76 (19) | 63 (16) | |
| TNBC | 289 (18) | 79 (20) | 81 (20) | 70 (18) | 59 (15) | |
| unknown/missing | 232 (15) | 49 (12) | 55 (14) | 50 (13) | 78 (20) | |
| **Grade** | | | | | | 0.10 |
| 1 (well) | 183 (12) | 51 (13) | 34 (9) | 46 (12) | 52 (13) | |

© 2023 Iyer HS et al. *JAMA Network Open.*

| | nSES (NCI Index) | | | | | |
|---|---|---|---|---|---|---|
| | **Total** | **Quartile 1** | **Quartile 2** | **Quartile 3** | **Quartile 4** | ***P*[a]** |
| **N (%)** | 1,575 | 394 | 396 | 391 | 394 | |
| 2 (moderate) | 610 (39) | 153 (39) | 162 (41) | 145 (37) | 150 (38) | |
| 3 (poor) | 695 (44) | 178 (45) | 172 (43) | 181 (46) | 164 (42) | |
| Unknown | 87 (6) | 12 (3) | 28 (7) | 19 (5) | 28 (7) | |
| **Stage** | | | | | | 0.011 |
| 0 | 280 (18) | 62 (16) | 70 (18) | 59 (15) | 89 (23) | |
| I | 574 (36) | 123 (31) | 144 (36) | 157 (40) | 150 (38) | |
| II | 504 (32) | 148 (38) | 134 (34) | 114 (29) | 108 (27) | |
| III | 170 (11) | 42 (11) | 38 (10) | 51 (13) | 39 (10) | |
| IV | 40 (3) | 15 (4) | 9 (2) | 9 (2) | 7 (2) | |
| Unknown | 7 (0) | 4 (1) | 1 (0) | 1 (0) | 1 (0) | |
| **ER-negative** | 450 (29) | 114 (29) | 119 (30) | 108 (28) | 109 (28) | 0.85 |
| **Treatment** | | | | | | |
| **Surgical treatment** | | | | | | 0.19 |
| No surgery | 51 (3) | 16 (4) | 11 (3) | 13 (3) | 11 (3) | |
| Lumpectomy | 791 (50) | 184 (47) | 186 (47) | 206 (53) | 215 (55) | |
| mastectomy | 733 (47) | 194 (49) | 199 (50) | 172 (44) | 168 (43) | |
| **Chemotherapy** | 864 (55) | 237 (60) | 231 (58) | 203 (52) | 193 (49) | 0.0041 |
| **Radiation therapy** | 1075 (68) | 257 (65) | 269 (68) | 272 (70) | 277 (70) | 0.43 |
| **Endocrine therapy** | | | | | | 0.39 |
| No | 573 (36) | 144 (37) | 158 (40) | 133 (34) | 138 (35) | |
| Yes | 1001 (64) | 250 (63) | 238 (60) | 257 (66) | 256 (65) | |
| Unknown/missing | 1 (0) | 0 (0) | 0 (0) | 1 (0) | 0 (0) | |

Abbreviations: ER=Estrogen Receptor, nSES=Neighborhood Socioeconomic Status, TNBC=Triple Negative Breast Cancer. [a]Wilcoxon or Kruskal-Wallis non-parametric tests to compare medians across quartiles of African ancestry; Chi-squared test of independence to compare prevalence.

© 2023 Iyer HS et al. *JAMA Network Open.*

**eTable 2. Hazard Ratios for Associations Between Clinical Predictors of Mortality in Breast Cancer Survivors, Stratified by African Ancestry and Neighborhood Socioeconomic Status in the Women's Circle of Health Follow-up Study, New Jersey, 2006-2020**

| | African ancestry | | | nSES (NCI Index) | | |
|---|---|---|---|---|---|---|
| | <85% | ≥85% | | ≥9219 | <9219 | |
| | aHR (95% CI) | aHR (95% CI) | $P_{het}$ | aHR (95% CI) | aHR (95% CI) | $P_{het}$ |
| **All-cause mortality**[a] | | | | | | |
| **Subtype** | | | 0.80 | | | 0.57 |
| Luminal | Ref | 0.96 (0.69, 1.33) | | Ref | 1.06 (0.75, 1.49) | |
| Her2+ | 1.19 (0.75, 1.90) | 1.39 (0.93, 2.07) | | 1.23 (0.76, 2.01) | 1.49 (0.98, 2.26) | |
| TNBC | 1.44 (0.93, 2.24) | 1.55 (0.99, 2.43) | | 1.78 (1.13, 2.80) | 1.46 (0.95, 2.22) | |
| **Grade** | | | 0.85 | | | 0.72 |
| 1 (well) | Ref | 0.82 (0.31, 2.13) | | Ref | 1.37 (0.53, 3.53) | |
| 2 (moderate) | 1.62 (0.81, 3.21) | 1.60 (0.81, 3.13) | | 2.20 (0.99, 4.86) | 2.05 (0.93, 4.50) | |
| 3 (poor) | 2.10 (1.05, 4.18) | 2.26 (1.14, 4.46) | | 2.78 (1.24, 6.25) | 2.94 (1.35, 6.37) | |
| **Stage** | | | 0.46 | | | 0.23 |
| 0/I | Ref | 1.54 (0.51, 4.60) | | Ref | 0.60 (0.20, 1.76) | |
| II | 2.72 (1.08, 6.89) | 3.36 (1.33, 8.47) | | 2.11 (0.99, 4.53) | 1.68 (0.79, 3.57) | |
| III/IV | 8.81 (3.69, 21.05) | 8.19 (3.42, 19.64) | | 4.74 (2.29, 9.83) | 5.70 (2.80, 11.59) | |
| **ER Status** | | | 0.43 | | | 0.99 |
| Positive | Ref | 0.98 (0.72, 1.32) | | Ref | 1.06 (0.78, 1.43) | |
| Negative | 1.30 (0.90, 1.89) | 1.56 (1.08, 2.25) | | 1.46 (0.99, 2.15) | 1.54 (1.08, 2.19) | |
| **Breast Cancer-specific Mortality**[a,b] | | | | | | |
| **Subtype** | | | 0.40 | | | 0.8 |
| Luminal | Ref | 0.79 (0.49, 1.28) | | Ref | 1.22 (0.76, 1.96) | |
| Her2+ | 1.25 (0.69, 2.27) | 1.56 (0.88, 2.74) | | 1.71 (0.96, 3.05) | 1.77 (0.96, 3.25) | |
| TNBC | 1.63 (0.95, 2.80) | 1.96 (1.17, 3.30) | | 2.27 (1.37, 3.78) | 2.16 (1.19, 3.93) | |
| **Grade** | | | 0.93 | | | 0.15 |
| 1/2 (well/moderate) | Ref | 0.95 (0.56, 1.61) | | Ref | 1.40 (0.83, 2.35) | |
| 3 (poor) | 1.69 (1.05, 2.74) | 1.66 (1.02, 2.71) | | 2.16 (1.37, 3.4) | 1.85 (1.12, 3.04) | |
| **Stage** | | | 0.11 | | | 0.0019 |
| 0/I | Ref | 1.91 (0.77, 4.77) | | Ref | 6.05 (1.81, 20.28) | |
| II-IV | 12.57 (5.69, 27.75) | 10.91 (4.89, 24.33) | | 29.74 (9.68, 91.33) | 24.81 (7.92, 77.71) | |
| **ER Status** | | | 0.24 | | | 0.4 |
| Positive | Ref | 0.85 (0.56, 1.30) | | Ref | 1.15 (0.76, 1.74) | |
| Negative | 1.61 (1.02, 2.56) | 2.01 (1.27, 3.17) | | 2.23 (1.45, 3.45) | 1.94 (1.19, 3.18) | |

© 2023 Iyer HS et al. *JAMA Network Open.*

Models were fit using robust (sandwich) errors to account for census tract-level clustering. [a]Models adjusted for age at diagnosis, interview year, education, mother's education, household income, insurance, and marital status. [b]Models fit using Fine-Gray competing risks proportional hazards models. Observations with non-missing clinical information were retained (n=1343 for subtype, n=1488 for grade, n=1568 for stage, n=1575 for ER)

© 2023 Iyer HS et al. *JAMA Network Open.*

**eTable 3. Odds Ratios for Associations Between African Ancestry and Tumor Subtypes Among Black Women With Breast Cancer, Restricted to High Quality Geocodes, Invasive Breast Cancer, US-Born, New Jersey, 2006-2020**

| | Continuous[e] | Quartile 1 | Quartile 2 | Quartile 3 | Quartile 4 | |
|---|---|---|---|---|---|---|
| | aOR (95% CI) | aOR (95% CI) | aOR (95% CI) | aOR (95% CI) | aOR (95% CI) | $P_{trend}$ |
| **$ER^-$ Breast Cancer (n=1051)** | | | | | | |
| African ancestry | | | | | | |
| $ER^-$ cases/$ER^+$ cases | | 79/190 | 99/195 | 80/201 | 76/131 | |
| Age[a] | 1.07 (0.95, 1.20) | Ref | 1.24 (0.87, 1.75) | 0.98 (0.68, 1.41) | 1.51 (1.04, 2.18) | 0.08 |
| Age + Ind SES[b] | 1.07 (0.94, 1.21) | Ref | 1.21 (0.85, 1.73) | 0.96 (0.66, 1.40) | 1.51 (1.03, 2.21) | 0.13 |
| Age + Ind SES + Lifestyles[c] | 1.07 (0.95, 1.22) | Ref | 1.24 (0.87, 1.77) | 0.96 (0.65, 1.41) | 1.54 (1.05, 2.27) | 0.09 |
| Age + Ind SES + Lifestyles + nSES[d] | 1.07 (0.95, 1.22) | Ref | 1.24 (0.87, 1.77) | 0.96 (0.65, 1.41) | 1.54 (1.05, 2.27) | 0.14 |
| **TNBC vs Luminal Breast Cancer (n=826)** | | | | | | |
| African ancestry | | | | | | |
| TNBC cases/Luminal cases | | 53/171 | 68/149 | 57/165 | 55/108 | |
| Age[a] | 1.14 (0.99, 1.31) | Ref | 1.50 (0.99, 2.27) | 1.14 (0.74, 1.75) | 1.83 (1.18, 2.84) | 0.03 |
| Age + Ind SES[b] | 1.13 (0.97, 1.30) | Ref | 1.48 (0.97, 2.24) | 1.07 (0.68, 1.66) | 1.81 (1.15, 2.85) | 0.05 |
| Age + Ind SES + Lifestyles[c] | 1.13 (0.97, 1.31) | Ref | 1.50 (0.99, 2.29) | 1.05 (0.67, 1.67) | 1.81 (1.14, 2.88) | 0.06 |
| Age + Ind SES + Lifestyles + nSES[d] | 1.13 (0.97, 1.31) | Ref | 1.50 (0.99, 2.29) | 1.05 (0.67, 1.67) | 1.81 (1.14, 2.87) | 0.06 |

Models were fit using robust (sandwich) errors to account for census tract-level clustering. Models sequentially adjusted for [a]age at diagnosis and interview year, [b]education, mother's education, household income, insurance, and marital status, [c]history of hypertension, history of diabetes, smoking, and body mass index, [d]nSES (interquartile range).
[e]Continuous measures are scaled to 10 percentage point increase for African ancestry, and interquartile range increase for nSES

© 2023 Iyer HS et al. *JAMA Network Open.*

**eTable 4. Hazard Ratios for Associations Between African Ancestry, Neighborhood Socioeconomic Status, and Among Black Women With Breast Cancer, Restricted to High Quality Geocodes, Invasive Breast Cancer, US-born (n=1,051), New Jersey, 2006-2020**

| | Continuous[g] | Quartile 1 | Quartile 2 | Quartile 3 | Quartile 4 | |
|---|---|---|---|---|---|---|
| | aHR (95% CI) | aHR (95% CI) | aHR (95% CI) | aHR (95% CI) | aHR (95% CI) | $P_{trend}$ |
| **All-cause mortality** | | | | | | |
| African ancestry | | | | | | |
| Deaths/person-years | | 51/1,973 | 64/2,065 | 61/1,937 | 48/1,512 | |
| Age[a] | 1.03 (0.92, 1.16) | Ref | 1.22 (0.83, 1.81) | 1.24 (0.85, 1.81) | 1.15 (0.78, 1.71) | 0.36 |
| Age + Ind SES[b] | 1.01 (0.90, 1.13) | Ref | 1.20 (0.81, 1.77) | 1.15 (0.79, 1.70) | 1.07 (0.72, 1.60) | 0.65 |
| Age + Ind SES + Lifestyles[c] | 1.02 (0.91, 1.15) | Ref | 1.16 (0.77, 1.75) | 1.27 (0.86, 1.87) | 1.06 (0.70, 1.61) | 0.56 |
| Age + Ind SES + Lifestyles + Treatment[d] | 1.03 (0.92, 1.15) | Ref | 1.25 (0.83, 1.89) | 1.29 (0.87, 1.92) | 1.10 (0.73, 1.66) | 0.46 |
| Age + Ind SES + Lifestyles + Treatment + nSES[e] | 1.03 (0.92, 1.15) | Ref | 1.24 (0.82, 1.87) | 1.28 (0.86, 1.90) | 1.10 (0.73, 1.65) | 0.48 |
| nSES | | | | | | |
| Deaths/person-years | | 74/2,019 | 59/1,827 | 53/1,938 | 38/1,702 | |
| Age[a] | 0.75 (0.59, 0.95) | Ref | 0.88 (0.61, 1.28) | 0.75 (0.52, 1.08) | 0.62 (0.41, 0.93) | 0.02 |
| Age + Ind SES[b] | 0.85 (0.67, 1.09) | Ref | 0.94 (0.65, 1.36) | 0.90 (0.62, 1.30) | 0.75 (0.50, 1.15) | 0.18 |
| Age + Ind SES + Lifestyles[c] | 0.87 (0.67, 1.12) | Ref | 0.94 (0.64, 1.37) | 0.86 (0.59, 1.26) | 0.78 (0.51, 1.21) | 0.25 |
| Age + Ind SES + Lifestyles + Treatment[d] | 0.87 (0.67, 1.13) | Ref | 0.96 (0.66, 1.39) | 0.92 (0.62, 1.35) | 0.77 (0.50, 1.20) | 0.24 |
| Age + Ind SES + Lifestyles + Treatment + African ancestry[e] | 0.89 (0.69, 1.15) | Ref | 0.96 (0.67, 1.38) | 0.89 (0.61, 1.30) | 0.80 (0.52, 1.13) | 0.29 |
| **Breast Cancer-specific Mortality[f]** | | | | | | |
| African ancestry | | | | | | |
| Deaths/person-years | | 33/1,973 | 34/2,065 | 35/1,937 | 24/1,512 | |
| Age[a] | 0.98 (0.84, 1.16) | Ref | 0.95 (0.58, 1.54) | 1.02 (0.62, 1.66) | 0.95 (0.55, 1.63) | 0.92 |
| Age + Ind SES[b] | 0.96 (0.82, 1.12) | Ref | 0.90 (0.55, 1.47) | 0.94 (0.58, 1.53) | 0.87 (0.52, 1.49) | 0.65 |
| Age + Ind SES + Lifestyles[c] | 0.97 (0.83, 1.14) | Ref | 0.88 (0.52, 1.47) | 1.00 (0.61, 1.65) | 0.90 (0.52, 1.56) | 0.80 |
| Age + Ind SES + Lifestyles + Treatment[d] | 0.96 (0.82, 1.12) | Ref | 0.85 (0.50, 1.44) | 0.95 (0.57, 1.57) | 0.85 (0.49, 1.47) | 0.63 |
| Age + Ind SES + Lifestyles + Treatment + nSES[e] | 0.96 (0.82, 1.13) | Ref | 0.86 (0.51, 1.46) | 0.95 (0.58, 1.57) | 0.85 (0.49, 1.48) | 0.64 |
| nSES | | | | | | |
| Deaths/person-years | | 37/2,019 | 35/1,827 | 27/1,939 | 27/1,702 | |
| Age[a] | 0.86 (0.64, 1.15) | Ref | 1.02 (0.64, 1.62) | 0.77 (0.47, 1.26) | 0.88 (0.53, 1.44) | 0.49 |
| Age + Ind SES[b] | 1.02 (0.76, 1.38) | Ref | 1.10 (0.69, 1.76) | 0.93 (0.56, 1.54) | 1.16 (0.70, 1.93) | 0.68 |
| Age + Ind SES + Lifestyles[c] | 0.96 (0.82, 1.13) | Ref | 1.05 (0.77, 1.43) | 1.07 (0.66, 1.73) | 0.92 (0.55, 1.53) | 0.54 |
| Age + Ind SES + Lifestyles + Treatment[d] | 1.07 (0.78, 1.47) | Ref | 1.05 (0.65, 1.69) | 0.97 (0.57, 1.65) | 1.19 (0.70, 2.04) | 0.57 |
| Age + Ind SES + Lifestyles + Treatment + African ancestry[e] | 1.07 (0.78, 1.46) | Ref | 1.05 (0.65, 1.69) | 0.97 (0.57, 1.65) | 1.18 (0.69, 2.03) | 0.59 |

Models were fit using robust (sandwich) errors to account for census tract-level clustering. Models sequentially adjusted for [a]age at diagnosis and interview year, [b]education, mother's education, household income, insurance, and marital status, [c]history of hypertension, history of diabetes, smoking, and body mass index and [d]molecular subtype and chemotherapy, and [e]nSES (interquartile range increase) or African ancestry (10 percentage point increase). [f]Models fit using Fine-Gray competing risks proportional hazards models. [g]Continuous measures are scaled to 10 percentage point increase for African ancestry, and interquartile range increase for nSES

© 2023 Iyer HS et al. *JAMA Network Open.*
