# Supplementary material for: Neighborhood Disadvantage, African Genetic Ancestry, Cancer Subtype, and Mortality Among Breast Cancer Survivors
Source: JAMA Netw Open. 2023 Aug 30;6(8):e2331295. doi: 10.1001/jamanetworkopen.2023.31295 (PMC10469269; doi:10.1001/jamanetworkopen.2023.31295)
Supplement: Supplement 2. — Data Sharing Statement [file jamanetwopen-e2331295-s002.pdf]

Data Sharing Statement

Iyer. Neighborhood Disadvantage, African Genetic Ancestry, Cancer Subtype, and Mortality Among Breast Cancer Survivors. *JAMA Netw Open*. Published August 30, 2023.
doi:10.1001/jamanetworkopen.2023.31295

Data
Data available: No

Additional Information
Explanation for why data not available: The de-identified data underlying this article can be shared upon approval of a data request form by the Women's Circle of Health Follow-up Study Scientific Committee and with appropriate human subjects approval and data transfer agreements. Inquiries can be directed to: wchstudy@cinj.rutgers.edu
